# The Therapeutics of Chronic Constipation

**Published:** 1870-09

**Authors:** 


### The Therapeutics of Chronic Constipation.

Dr. J. K. Spencer, of Bath, England, in a paper upon this subject contributed to the *Medical Times and Gazette*, says:

The plan which I propose comprises four therapeutics factors; (*a*) minute and frequent doses of watery extract of aloes, very rare-l yof extract of colocynth; (*b*) a dose of sulphate of iron (gr. jss or ij) always combined with each dose of the direct aperient; (*c*) regulation of the diet; (*d*) constitutional exercise. I have to write chiefly of factors (*a*) and (*b*). The quantity of extract of aloes, in all but extraordinary cases, should not exceed one grain. It is conveniently given in the form of a pill. With this pill there should always be mixed a dose of sulphate of iron varying from one to three grains; this is the essential point of the treatment. any other tonic of the neurotic kind cannot supply the place of iron; for the purpose I am now relating, iron is not only *facile princeps*, but is not interchangeable by anything else. Extract of nux vomica may be added, if the prescriber pleases, as an ornamental appendage or as a means of blending the other constituents together; and belladonna is a remedy of definite auxiliary power, but both these drugs, *quoad* constipation of the bowels, are uncertain or unsatisfactory, and rarely do permanent good. I begin, then, by desiring an adult patient to take a pill composed as above three times a day, immediately after the principal meals. He is cautioned that at first there will be probably no apparent effect, and that two or even three days may pass before any medicinal evacuation of the bowels takes place, perhaps even then difficult and discomforting. But within the next forty-eight hours there will be most likely an evacuation of the bowels once, or possibly twice in the day; *but nothing approaching to purgation ought ever to be permitted*, and therefore the patient must be instructed, on the occurence of the first loose motion, to withhold a pill, and to take only one in the morning and one in the evening. He then continues for a time his morning and evening pill, and is pleased to discover that so slender a medicament has such a decided effect. Not improbably, at the end of another week or fortnight he is compelled, by the same reason as before, to drop another pill, and the same result is now brought about by one pill daily, as was originally produced by three pills. Within another month, he may reduce his allowance of medicine to a single pill once or twice a week; and, finally, his whole scheme of medical treatment becomes merely preventive in its design and scope, and he takes a pill occasionally for the sake of maintaining health and warding off old troubles.—*N. Y. Med. Jour.*

